# Delay-Informed Intelligent Formation Control for UAV-Assisted IoT Application

**DOI:** 10.3390/s23136190

**Published:** 2023-07-06

**Authors:** Lihan Liu, Mengjiao Xu, Zhuwei Wang, Chao Fang, Zhensong Li, Meng Li, Yang Sun, Huamin Chen

**Affiliations:** 1 School of Statistics and Data Science, Beijing Wuzi University, Beijing 101149, China; liulihan@bwu.edu.cn; 2 Faculty of Information Technology, Beijing University of Technology, Beijing 100124, China; xumengjiao@emails.bjut.edu.cn (M.X.); fangchao@bjut.edu.cn (C.F.); limeng720@bjut.edu.cn (M.L.); sunyang@bjut.edu.cn (Y.S.); chenhuamin@bjut.edu.cn (H.C.); 3 Purple Mountain Laboratory: Networking, Communications and Security, Nanjing 210096, China; 4 School of Information and Communication Engineering, Beijing Information Science and Technology University, Beijing 100101, China; lizhensong@bistu.edu.cn

**Keywords:** surveillance, formation control, intelligent control strategy, time delay, dynamic leading velocity

## Abstract

Multiple unmanned aerial vehicles (UAVs) have a greater potential to be widely used in UAV-assisted IoT applications. UAV formation, as an effective way to improve surveillance and security, has been extensively of concern. The leader–follower approach is efficient for UAV formation, as the whole formation system needs to find only the leader’s trajectory. This paper studies the leader–follower surveillance system. Owing to different scenarios and assignments, the leading velocity is dynamic. The inevitable communication time delays resulting from information sending, communicating and receiving process bring challenges in the design of real-time UAV formation control. In this paper, the design of UAV formation tracking based on deep reinforcement learning (DRL) is investigated for high mobility scenarios in the presence of communication delay. To be more specific, the optimization UAV formation problem is firstly formulated to be a state error minimization problem by using the quadratic cost function when the communication delay is considered. Then, the delay-informed Markov decision process (DIMDP) is developed by including the previous actions in order to compensate the performance degradation induced by the time delay. Subsequently, an extended-delay informed deep deterministic policy gradient (DIDDPG) algorithm is proposed. Finally, some issues, such as computational complexity analysis and the effect of the time delay are discussed, and then the proposed intelligent algorithm is further extended to the arbitrary communication delay case. Numerical experiments demonstrate that the proposed DIDDPG algorithm can significantly alleviate the performance degradation caused by time delays.

## 1. Introduction

Recently, the development of unmanned aerial vehicles (UAVs) has brought many benefits in UAV-assisted application fields, such as surveillance, rescue, reconnaissance and search [1,2]. The UAV formation control, driving each vehicle to reach the prescribed constraint on its own states through generating appropriate control commands, significantly expands the potential applications and opens up new possibilities for UAVs. For example, a group of UAVs could expand the fields of view when executing assignment.

A task of cooperative surveillance is considered in this paper. The target is to guide a group of UAVs equipped with cameras to fly over an urban area (possibly hostile) to provide complete surveillance coverage in an optimal manner [3]. Considering the limitation of batteries, leader–follower units are introduced to make a group of UAVs fly with a formation in order to improve the efficiency and expand the field of surveillance. In this paper, the leader is assigned to make the flying strategy, like the flying velocity and trajectory position, depend on the environment information transmitted by wireless sensor agent networks (WSANs). Followers focus on tracking with a dynamic leader and keep a desired cooperation formation. In this paper, we focus on the design of the controller to make followers achieve the desired cooperation formation while tracking a dynamic leader.

However, UAVs are underactuated systems constrained by high mobility and serious disturbances [4]. Therefore, it becomes a great challenge to address the robust formation controller design problem to enable UAVs to achieve the desired cooperation formation. The traditional optimal formation control methods, such as the nonlinear model predictive control (see [5,6]), and nonlinear PID control (see [7,8]), are proposed to alleviate the degradation of control stability attributed to the external disturbances and uncertainties in the UAV formation. These approaches can generally be regarded as a cost function minimization problem defined by a set of UAV states and control actions. Unfortunately, the above methods often fail to generalize to the wider range of application scenarios due to the highly dynamic and time-varying features of UAVs.

Existing approaches have been proposed to overcome the limitations of traditional formation control algorithms, among which the highest potential one is reinforcement learning (RL) [9]. In fact, RL is a classical learning method to address the sequential decision-making problem within the Markov decision process (MDP). At each step, the agent interacts with the environment and derives a reward. After exploration and training, the control policy gradually achieves the optimal trategy. By using the framework of MDP, RL is a typical algorithm developed in the control field originally for optimal stochastic control under uncertainty [10]. Different from the classical rule-based optimization methods, RL learns intelligently in each step, interacting with the environment to derive approximate optimal model parameters.

In order to improve the learning ability of RL, deep reinforcement learning (DRL), integrating the benefits of both RL and deep neural networks (DNNs), has been proposed. DRL can efficiently handle a much more complicated state space and dynamic environment, and achieve superior performance for game-playing tasks [11,12,13]. DRL has become a research hotspot in the field of UAV control, such as the outer-loop control (formation maintenance, navigation [14], path planning [15]) and inner-loop control (altitude [16]). In DRL, the deep Q learning (DQN) technique is employed to reduce the correlation among successive experience samples by using an experience replay buffer. Nevertheless, DQN can only deal with a limited action space, while the UAV formation control is a continuous control process with an unlimited action space. Then, the actor–critic method is further developed for continuous control action [9]. Based on the actor–critic framework, the deep deterministic policy gradient (DDPG) algorithm, which takes advantage of the DQN experience replay and dual network structure to enhance the deterministic policy gradient (DPG) algorithm, has been used comprehensively for continuous agent control, and its feasibility has been validated in many potential scenarios, such as autonomous driving, (longitudinal see [17], mixed-autonomy see [18]), UAV (navigation see [19], motion control see [20]), etc.

Formation control requires continual and real-time information exchange. At each time interval, environment information should be exchanged (i.e., sent or received) by sensor nodes through the WSANs, which typically suffers from a series of issues, such as network topology, network traffic and system resource limitations, resulting in inevitable network-induced time delays. In our surveillance study, the leader collect the environment information through the sensor nodes spread in WSANs to make the flying strategy, including velocity and position. Then, the new flying strategy, including the velocity and position of the leader, is subsequently transmitted to the follower.

Considering the leader–follower units as an whole unit, this whole unit collects environment information through WSANs and produces action, like an agent in MDP. Consequently, the agent’s observations of its environment are not immediately available due to the quality of WSANs, and the time delay actually exists in the action selection and actuation of the agent in MDP. However, most existing DRL-based algorithm designs are restrained to synchronous systems with delay-free observations and action actuation [21,22,23]. Therefore, it is of great practical significance to investigate the intelligent UAV formation control considering the time delay constraint. In this paper, we propose a novel intelligent formation control algorithm to deal with the time delay issue in accordance with the model-based DDPG.

### 1.1. Related Works

The UAV formation control includes three typical types, such as formation generation and maintenance, formation shape maintenance and regeneration and formation maintenance while trajectory tracking [24]. Refs. [25,26,27,28,29] integrate these types into an optimal formation tracking problem. Although these works have the capability to meet the formation maintenance requirement, they fail to deal with much more complex environments because the algorithm parameters cannot be intelligently adjusted according to the dynamic feature of environments. Therefore, it is meaningful to introduce RL algorithms to UAV formation control.

Several new techniques are developed based on the DRL to address the UAV control problem. The DQN algorithm is employed in [30] for real-time UAV path planning. A double deep Q-network (DDQN) is further trained in [15] using the experience replay buffer in order to learn to generate the control policy according to time-varying scenario parameters for UAV. Li et al. [14] focus on the ground target tracking to solve the obstacle problem for UAV system using the improved DDPG. In [31], an end-to-end DRL model is developed for the indoor UAV target searching. Unfortunately, the research of DRL-based UAV formation maintenance is still not enough. In addition, these studies have ignored the effect of the time delay issue, which is an inherent feature in actual UAV formation.

Currently, the study of the RL-based algorithm design with delays is attracting more and more attention. For example, in the design of MDP, Walsh et al. [32] first directly increased the length of a sampling interval in order to achieve the agent’s action synchronization using the delayed observations, and then the authors further introduced the delayed actions to the state, which effectively compensates for the effect of time delay. Refs. [33,34,35,36,37,38] formally described the concept of delayed MDP, and demonstrated that the delayed MDP can be transformed into an equivalent standard MDP, and then it can be employed to formulate the delay-resolved RL framework to derive the near-optimal rewards interacting with the environments. In [39], a delay-aware MDP is proposed to address the continuous control task by increasing the state space with a sequence being executed in the next delay duration step. The interaction manner they proposed is motivated by applying an action buffer as an interval. The agent can obtain environment observation as well as the future sequences from the action buffer, and then determine its future action.

In general, the above methods can be divided into two types, one is that the state space of the learning agent is integrated with the delayed action, and the other is to learn a model of the underlying delay-free process to predict the control actions for future states. Motivated by the existing RL approaches with time delays, the design of UAV formation tracking based on deep reinforcement learning is further developed in our work to address the UAV formation problem in the presence of time delays. In fact, there are few works to address the influence of time delays on intelligent UAV formation in highly dynamic scenarios. However, considering the actual real-time formation control, the time delay is an inherent feature that needs to be studied to improve the control stability.

### 1.2. Contribution

Due to the uncertainty of wireless communications, the information transmission in UAV formation control will suffer from time delays, which may lead to control instability and formation performance degradation, especially in the high dynamic applications [40,41,42]. Neither different from the intelligent algorithm in [43], which ignores the influence of time delay, nor different from traditional control methods, such as Artstein’s model reduction [44] and Smith predictor [45], which are restrained to be applied to much more complex and dynamic scenarios because of their limited intelligent adaptability, a delay-informed intelligent framework is proposed in the paper to address the UAV formation problem subject to time delays. The main contributions of our work are as follows:

- In order to regulate the UAV motion, the UAV formation model considering time delay is first established in discrete-time form based on the UAV error dynamics. Then, an optimization problem designed to minimize the quadratic cost function is formulated for the optimal formation control under time delays.
- According to the error dynamics and optimization formation control problem, a delay-informed MDP (DIMDP) framework is presented by including the previous control actions into the state and reward function. Then, a DRL-based algorithm is proposed to address DIMDP, and the classical DDPG algorithm is extended as a delay-informed DDPG (DIDDPG) algorithm to solve DIMDP.
- The computational complexity analysis and the effect of the time delay are discussed, and the proposed algorithm is further extended to the arbitrary communication delay case. Through the training results, the proposed DIDDPG for the UAV formation control can achieve better convergence and system performance.

The rest of this paper is organized as follows. The system model and UAV formation optimization problem are presented in Section 2. In Section 3, the environment model is established as DIMDP, and then the DIDDPG algorithm is proposed to solve DIMDP. Section 4 shows the simulation results, and Section 5 concludes our work.

## 2. System Modeling and Problem Formulation

In this section, by considering the time delay and dynamic leader velocity, the formation control model is first presented. Then, the cost function based on the discrete-time states errors is designed for the follower to reach the desired states. Finally, the optimization problem is formulated.

### 2.1. System Modeling

UAV formation can be applied to a multitude of security and surveillance areas. The pattern formation is crucial for multi-UAV formation control mechanisms while cautiously navigating the surveillance areas. The leader–follower formation is introduced to improve the efficiency for UAV formation, as the surveillance system needs to find only the leader’s trajectory.

In this paper, the UAV formation is divided into several leader–follower control units, with one UAV designated as the leader and the remaining UAVs are as followers. By realizing the tracking mission of each unit, the mission of the whole formation is realized. In the formation control process, wireless communication technology is used to complete the information collection and sharing through the WSAN. The leader can receive mission and formation information, and then use the received information to plan the trajectory and guide the direction of the entire formation. The controller regularly collects the position, speed and other status information of the leader and the follower, and calculates the state error of the follower, and then generates and transmits the control strategy to the follower actuator to ensure the stability of formation control. At the same time, communication delays, including leader-to-controller, controller-to-follower actuator, and information processing delays are introduced.

**Figure 1:**
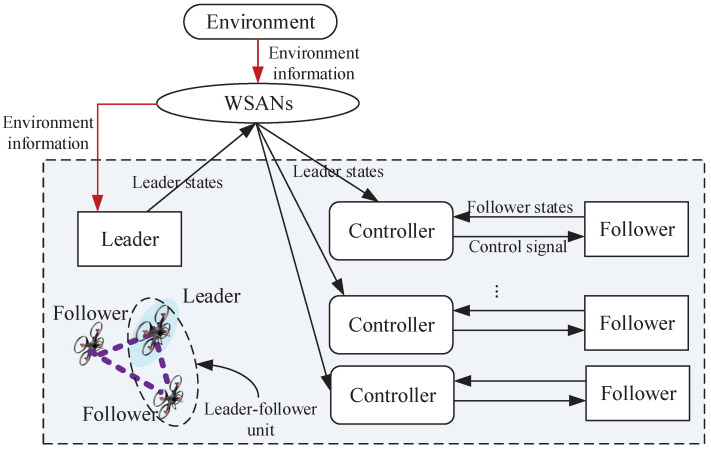
UAV formation system model.

**Figure 2:**
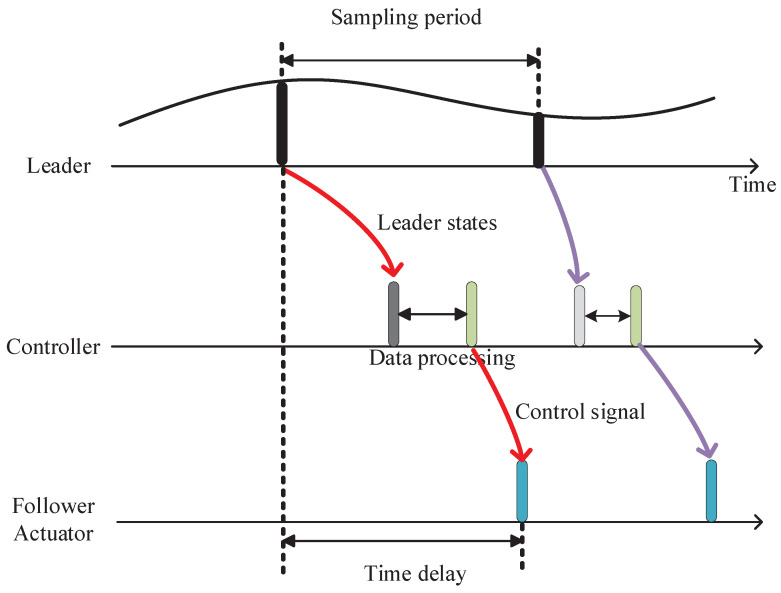
Timing diagram for the leader–follower formation control.

The considered formation control model and corresponding timing diagram are shown as Figure 1 and Figure 2, respectively. The leader is assigned to make the flying strategy, like the flying trajectory and speed, depend on the shared environment information, such as mission and formation information transmitted through the WSAN. The leader makes an appropriate strategy, such as acceleration, deceleration and hover, due to the relevant real-world scenarios and assignments. For example, the formation needs to change when encountering obstacles. Then, the updated formation state information is transmitted to the controllers through the WSAN. Therefore, the leader-to-controller delay is introduced. Once the formation information is collected, the controller can calculate and generate the control strategy, and then transmit it to the follower actuator to improve the formation control. Meanwhile, the controller-to-follower actuator delay and data processing delay are introduced.

In fact, the location of controller can be placed on the leader UAV or the follower UAV or the ground control center according to the real-world application scenarios. For example, in [21], an intelligent controller placed in the follower is proposed and it is testified that this approach is applicable in many applications, such as penetration and remote surveillance. Figure 2 is able to include all the delay cases, no matter where the controller is placed; due to this, Figure 2 shows a general case for communication delays of formation control. For example, when the controller is placed on the follower, the time delay from the controller to the follower actuator will be small or even negligible. In our work, the dynamic leading velocity and time delay are considered due to the complex environment and real-world application.

Considering a leader–follower unit, the kinematics of the follower is given by

(1) $$\begin{array}{cc} \; & \dot{p}\left(t\right)=v\left(t\right),\\ \; & \dot{v}\left(t\right)=c\left(t-\tau \left(t\right)\right), \end{array}$$

where $v\left(t\right)$ and $p\left(t\right)$ are the velocity and position of the follower, respectively, $c\left(t\right)$ denotes the acceleration of the follower (i.e., the control strategy), τ(t) is the time delay shown as in Figure 2, which accounts to the signal processing delay and the transmission latency from the leader to the controller and from the controller to the follower, and the time delay is typically assumed to be stochastic due to the quality of WSANs.

The model of desired states can be described as [46]

(2) $$\begin{array}{cc} \; & {\dot{p}}_{r}\left(t\right)=v_{r}\left(t\right),\\ \; & {\dot{v}}_{r}\left(t\right)=f_{r}\left(p_{r}\left(t\right),v_{r}\left(t\right)\right), \end{array}$$

where $v_{r}\left(t\right)$ and $p_{r}\left(t\right)$ are the expected velocity and position, respectively, which are determined by the state of the leader, and $f_{r}\left(p_{r}\left(t\right),v_{r}\left(t\right)\right)$ denotes the time-varying acceleration of the leader.

The objective of the follower is to maintain the formation and track the leader. Define the state errors of the follower as follows:

(3) $$\begin{array}{cc} \; & \Delta p\left(t\right)=p\left(t\right)-p_{r}\left(t\right),\\ \; & \Delta v\left(t\right)=v\left(t\right)-v_{r}\left(t\right). \end{array}$$

Then, based on Formulas (1)–(3), the relationship among state errors can be deduced as

(4) $$\begin{array}{cc} \; & \Delta \dot{p}\left(t\right)=\dot{p}\left(t\right)-{\dot{p}}_{r}\left(t\right)=v\left(t\right)-v_{r}\left(t\right)=\Delta v\left(t\right),\\ \; & \Delta \dot{v}\left(t\right)=\dot{v}\left(t\right)-{\dot{v}}_{r}\left(t\right)=c\left(t-\tau (t)\right)-f_{r}\left(p_{r}\left(t\right),v_{r}\left(t\right)\right). \end{array}$$

which indicates that the differential of the position error presents the change in velocity, and the differential of the velocity error denotes the change in acceleration.

Note that τ(t) is a time-varying item due to the uncertainty of the transmission environment, and $f_{r}\left(p_{r}\left(t\right),v_{r}\left(t\right)\right)$ is an unknown item due to the dynamic feature of the leader acceleration.

### 2.2. Optimization Problem Formulation

Define $z\left(t\right)={\left[\Delta p^{x}\left(t\right),\;\Delta v^{x}\left(t\right),\;\Delta p^{y}\left(t\right),\;\Delta v^{y}\left(t\right),\;\Delta p^{z}\left(t\right),\;\Delta v^{z}\left(t\right)\right]}^{T}$ as the state vector, where the superscripts *x*, *y* and *z* represent the 3D information of state errors. Based on the stat error model (4), the follower dynamics can be expressed as follows:

(5) $$\begin{array}{c} \dot{z}\left(t\right)=Az\left(t\right)+B\left[c\left(t-\tau \left(t\right)\right)-f_{r}\left(p_{r}\left(t\right),v_{r}\left(t\right)\right)\right], \end{array}$$

where

$$A=\left[\begin{array}{ccc} \bar{A} & 0_{2\times 2} & 0_{2\times 2}\\ 0_{2\times 2} & \bar{A} & 0_{2\times 2}\\ 0_{2\times 2} & 0_{2\times 2} & \bar{A} \end{array}\right],$$

$$B=\left[\begin{array}{ccc} \bar{B} & 0_{2\times 1} & 0_{2\times 1}\\ 0_{2\times 1} & \bar{B} & 0_{2\times 1}\\ 0_{2\times 1} & 0_{2\times 1} & \bar{B} \end{array}\right],$$

that $0_{i\times j}$ is the zero matrix, and 

$$\bar{A}=\left[\begin{array}{cc} 0 & 1\\ 0 & 0 \end{array}\right],\;\bar{B}=\left[\begin{array}{c} 0\\ 1 \end{array}\right].$$

During each sampling interval, the controller receives the measurement state information, and then derived the control strategy to improve the formation control stability. Then, the corresponding discrete-time dynamics of the follower in the *j*-th sampling interval [jT,(j+1)T) is given by

(6) $$\begin{array}{c} z_{j+1}=Ez_{j}+D_{j}^{1}c_{j}+D_{j}^{2}c_{j-1}+G_{j}, \end{array}$$

where

$$\begin{array}{cc} & E=e^{AT},\;D_{j}^{1}=\int_{0}^{T-\tau_{j}}e^{At}dtB,\;D_{j}^{2}=\int_{T-\tau_{j}}^{T}e^{At}dtB,\\ & G_{j}=\int_{jT}^{(j+1)T}e^{A[(j+1)T-s]}f_{r}\left(p_{r}\left(s\right),v_{r}\left(s\right)\right)dsB, \end{array}$$

and $z_{j}$ and $\tau_{j}$ are the sampled values of $z\left(t\right)$ and τ(t) at time jT, respectively, and $c_{j}$ denotes the control signal relevant to the received state $z_{j}$.

Note that the time delay $\tau_{j}$ causes the time-varying feature of $D_{j}^{1}$ and $D_{j}^{2}$, and the dynamic leader movement also introduces the uncertain item $G_{j}$, which increases the difficulty for traditional algorithms to address these dynamic features. Additionally, in each sampling interval, the influence of the previous control signals is further introduced due to the time delays.

The objective of the follower is to minimize state errors. Therefore, the typical quadratic optimization problem for formation control can be formulated as [22]

(7) $$\begin{array}{cc} \; & \min_{\left\{c_{j}\right\}}\;E\left[z_{N}^{T}Pz_{N}+\sum_{j=0}^{N-1}\left(z_{j}^{T}Pz_{j}+c_{j}^{T}Qc_{j}\right)\right]\\ \; & \;\;s.t.\;z_{j+1}=Ez_{j}+D_{j}^{1}c_{j}+D_{j}^{2}c_{j-1}+G_{j}, \end{array}$$

where E denotes the expectation based on the stochastic natures of the leader movement and time delays, *P* and *Q* are system parameters, and *N* is the finite time horizon.

## 3. DIDDPG Algorithm for Formation Control

In this section, the DIMDP framework is first presented, and then the environment model which maps the system model to the interaction environment of DIMDP is formulated. Additionally, a DIDDPG algorithm for the UAV formation controller design is proposed.

### 3.1. DIMDP-Based Environmental Model

**Figure 3:**
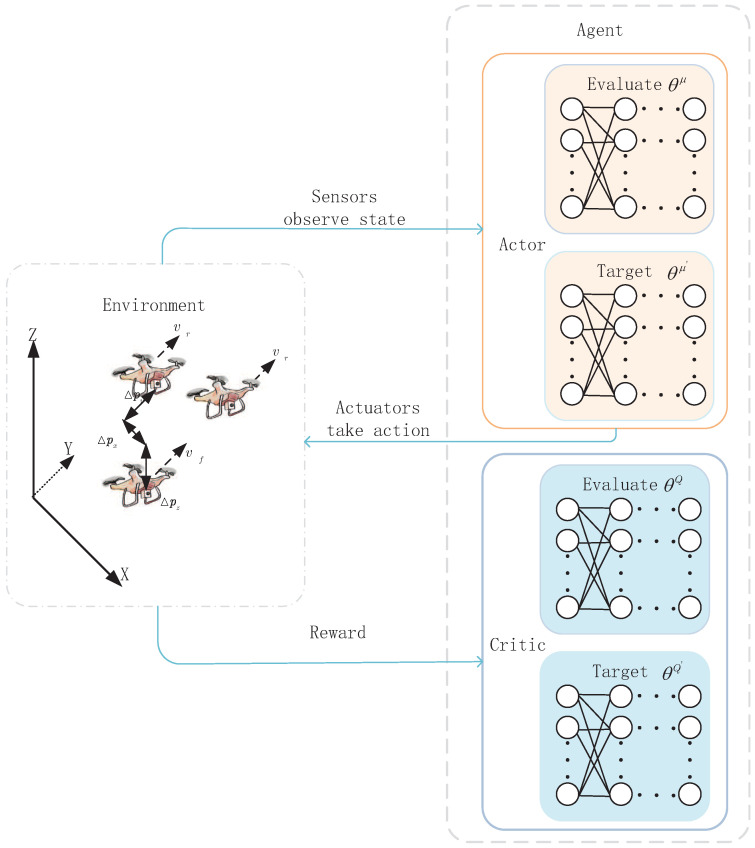
The framework of MDP for the leader–follower formulation.

The framework of MDP for the leader–follower formulation is shown as in Figure 3. At each time slot, based on the observed current UAV states from the environment, the action is generated and executed according to the action policy. Then, the new state is updated by the state transition function, and the corresponding reward is returned to the agent. In the framework of MDP, the actor0-critic structure, integrating the advantages of the policy search method with the value function learn, is used.

Considering the fact that time delay is an inevitably negative factor to the real-time control, in order to address the optimization formation problem in (7), the MDP framework associated with the time delay needs to be formulated. In fact, the basic MDP framework typically assumes that the system’s current states are always available to the agent and the agent always takes relevant actions immediately. However, these assumptions are not appropriate for the optimization formulation problem because of the time delay. How to integrate the effect of time delay into the MDP framework design is the key issue. Therefore, DIMDP, the standard MDP extension with time delay, is proposed, in which the agent interacts with the environment, and the environment is influenced by the delayed control strategies (i.e., the delayed actions). Below, the detailed definitions of the state space, action space, state transition function and reward function for the DIMDP are given.

**Figure 4:**
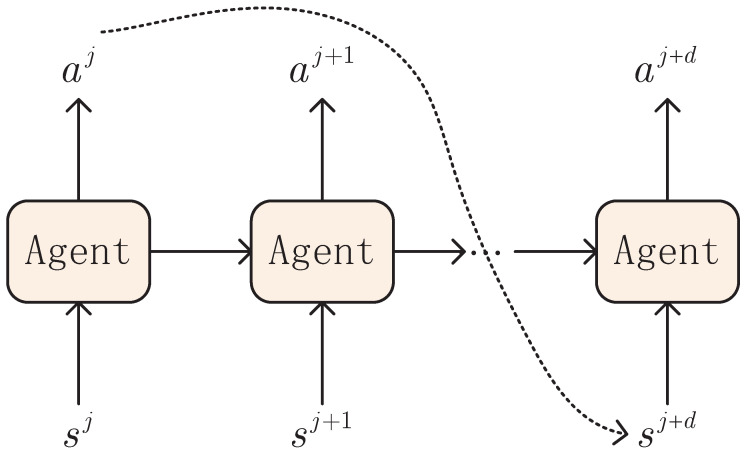
Delay-informed MDP.

(1) State: Referring to the leader–follower UAV formation, several factors, including the action of the follower and the error states between the leader and the follower, are considered. As shown in (4), the state errors of the follower are determined by the position and velocity errors. From the discrete-time dynamics (6), the effect of the previous control strategy $c_{j-1}$ is also attributed to the time delay as shown in Figure 4. Therefore, the state in the *j*-th sampling interval is defined as
  (8) $$\begin{array}{cc} s_{j} & =\left[{z_{j}}^{T},\;{c_{j-1}}^{T}\right]\\ \; & ={\left[\Delta p_{j}^{x},\;\Delta p_{j}^{y},\;\Delta p_{j}^{z},\;\Delta v_{j}^{x},\;\Delta v_{j}^{y},\;\Delta v_{j}^{z},\;\Delta a_{j-1}^{x},\;\Delta a_{j-1}^{y},\;\Delta a_{j-1}^{z}\right]}^{T}. \end{array}$$
  In (8), the updated state error information and local previous control strategy information are extracted to represent the environment state to regulate the follower UAV tracking. In particular, the previous control strategy is used to compensate for the effects of the time delay.
(2) Action: The decision action is given by
  (9) $$\begin{array}{c} a_{j}=c_{j}^{T}, \end{array}$$
  where $a_{j}$ is actually the acceleration policy of the follower UAV, which is a continuous value, and  we have
  (10) $$c_{\min}\leq a_{j}\leq c_{\max},$$
  which indicates that the action is constrained by boundary values.
(3) State transition function: The state transition function can be determined according to the discrete-time dynamics of the follower in (6) as follows:
  (11) $$s_{j+1}=s_{j}F_{j}+a_{j}H_{j}+\left[G_{j}^{T},0_{3\times 1}\right],$$
  where
  $$F_{j}=\left[\begin{array}{cc} E & 0_{3\times 6}\\ D_{j}^{2} & 0_{3\times 3} \end{array}\right],\;H_{j}=\left[\begin{array}{c} D_{j}^{1}\\ I_{3\times 3} \end{array}\right].$$
(4) Reward function: The reward is used to evaluate the performance of the action, and then the follower can intelligently learn to derive the proper control strategy to maintain the formation tracking. The reward function can be designed as the opposite of the cost function in terms of the optimization problem in (7) as follows:
  (12) $$r_{j}=-s_{j}\bar{P}s_{j}^{T}-a_{j+1}Qa_{j+1}^{T},$$
  where
  $$\bar{P}=\left[\begin{array}{cc} P & 0_{3\times 6}\\ 0_{6\times 3} & 0_{3\times 3} \end{array}\right].$$
  In fact, the closer the follower’s states are to the desired ones, the greater the reward. It is significant that, based on the well-designed reward function, the follower can rapidly achieve the desired position and velocity by continuously adjusting the action in order to acquire the maximum long-term cumulative rewards, which is formulated as a finite horizon *N* item by
  (13) $$\begin{array}{c} G_{N}=-\sum_{j=0}^{N-1}\gamma^{j}\left(s_{j}\bar{P}s_{j}^{T}+a_{j}Qa_{j}^{T}\right), \end{array}$$
  where γ is a discount factor.

### 3.2. DIDDPG UAV Formation Algorithm

In this section, we employ the DDPG method with the DIMDP definitions, and then a model-based DIDDPG algorithm for the continuous UAV formation control is proposed.

**Figure 5:**
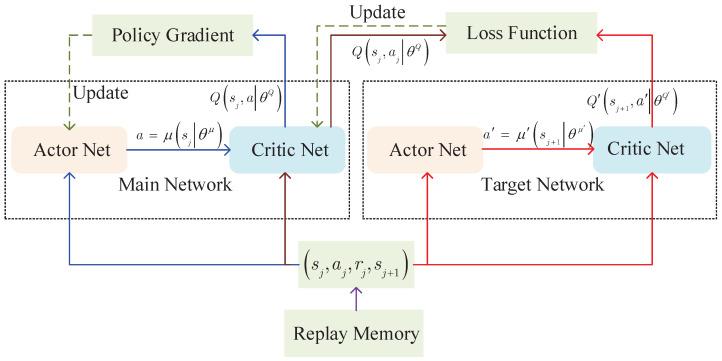
Framework of DIDDPG algorithm.

The framework of DIDDPG is presented as in Figure 5. The main network includes two parts (i.e., critic network and actor network). The actor network $\mu \left(s|\theta^{\mu }\right)$ builds a mapping from states to actions, and the main policy is generated, while the critic network $Q\left(s,a|\theta^{Q}\right)$ estimates the action value, where $\theta^{Q}$ and $\theta^{\mu }$ are parameters of the critic network and actor network, respectively. The target network is employed for the actor–critic architecture to acquire a stable target *Q* value. The parameters of target network $\mu^{\prime }\left(s|\theta^{\mu^{\prime }}\right)$ and target critic $Q^{\prime }\left(\left.s_{j+1},a^{\prime }\right|\theta^{Q^{\prime }}\right)$ update based on the main network parameters.

In each time slot *j*, the online actor network generates the corresponding action policy $\mu \left(s_{j}|\theta^{\mu }\right)$ based on state $s_{j}$. After executing the action $a_{j}=\mu \left(s_{j}|\theta^{\mu }\right)+\eta$ (η is an additional random noise to ensure the effective exploration), the next state $s_{j+1}$ can be updated based on (11), and the corresponding reward $r_{j}$ can be obtained according to (12). Then, the transition $\left(s_{j},a_{j},s_{j+1},r_{j}\right)$ is stored as a sample in the experience replay memory buffer. Repeating this process based on the closed loop control, enough training data can be generated by interacting with the environment. While training the networks, the mini-batch of *K* experience samples are randomly selected from the experience replay memory buffer in order to reduce the correlation among samples that the training efficiency can be improved.

By minimizing the loss function $L(\theta^{Q})$, typically defined as a mean quadratic error function, the main critic network can update the parameter $\theta^{Q}$ using the gradient descent method:

(14) $$\begin{array}{c} L\left(\theta^{Q}\right)=\frac{1}{K}\sum_{j=0}^{K-1}{\left(Q\left(s_{j},a_{j}|\theta^{Q}\right)-y_{j}\right)}^{2}, \end{array}$$

where $Q(s_{j},a_{j}|\theta^{Q})$ represents the current *Q* value generated by the output of main critic network based on action $a_{j}$ and state $s_{j}$, and $y_{j}$ is the target *Q* value given by

(15) $$y_{j}=r_{j}+\gamma Q^{\prime }\left(s_{j+1},\mu^{\prime }\left(s_{j+1}|\theta^{\mu^{\prime }}\right)|\theta^{Q^{\prime }}\right).$$

In (15), $\mu^{\prime }\left(s_{j+1}|\theta^{\mu^{\prime }}\right)$ and $Q^{\prime }\left(s_{j+1},\mu^{\prime }\left(s_{j+1}|\theta^{\mu^{\prime }}\right)|\theta^{Q^{\prime }}\right)$ denote the next action policy and next *Q* value derived from the target actor and critic networks, respectively.

Then, the main actor network updates the parameter $\theta^{\mu }$ by the policy’s gradient algorithm as [47]

(16) $$\nabla_{\theta^{\mu }}J\approx \frac{1}{K}\sum_{j=0}^{K-1}\left[\nabla_{a}Q\left(s,a|\theta^{Q}\right){|}_{s=s_{j},a=\mu \left(s_{j}\right)}\nabla_{\theta^{\mu }}\mu \left(s|\theta^{\mu }\right){|}_{s_{j}}\right],$$

The updating gradient of the policy helps to improve the possibility of choosing a better action. Then, the DIDDPG softly updates the target networks as

(17) $$\begin{array}{c} \theta^{Q^{\prime }}\leftarrow \delta \theta^{Q}+\left(1-\delta \right)\theta^{Q^{\prime }},\\ \theta^{\mu^{\prime }}\leftarrow \delta \theta^{\mu }+\left(1-\delta \right)\theta^{\mu^{\prime }}. \end{array}$$

and here, δ is a small constant.

After training, the parameters $\theta^{\mu^{*}}$ will converge, and then the optimal formation control strategy for the follower is derived as

(18) $$a^{*}=\mu \left(s|\theta^{\mu^{*}}\right).$$

The detailed DIDDPG-based UAV formation algorithm is presented as Algorithm 1.
**Algorithm 1** DIDDPG-based UAV formation algorithm.  1:Initialize system parameters $\bar{P},F_{j},H_{j},D_{j}^{1},D_{j}^{2}$ and the replay memory buffer *R*.  2:Randomly initialize $\theta^{\mu }$, $\theta^{Q}$, $\mu^{\prime }$ and $Q^{\prime }$.  3:Initialize online actor and critic networks $\mu \left(s\left|\theta^{\mu }\right.\right)$ and $Q\left(s,a\left|\theta^{Q}\right.\right)$, respectively.  4:**for** episode = 0:1:N−1 **do**  5:   Initialize the random noise ω and state $s_{0}$.  6:   **for** j=0:1:M−1 **do**  7:     Update the action $a_{j}=\mu \left(s_{j}\left|\theta^{\mu }\right.\right)+\omega$.  8:     Update the next state $s_{j+1}$ based on (11) that $s_{j+1}=s_{j}F_{j}+a_{j}H_{j}+\left[G_{j}^{T},0_{3\times 1}\right]$.  9:     Derive the reward $r_{j}$ by (12) that $r_{j}=-s_{j}\bar{P}s_{j}^{T}-a_{j+1}Qa_{j+1}^{T}$.10:     Store transition $\left(s_{j},a_{j},r_{j},s_{j+1}\right)$ in *R*.11:     Randomly Select a mini-batch of *K* experience samples $\left(s_{j},a_{j},r_{j},s_{j+1}\right)$ from *R*.12:     Update target *Q* value based on (15) that $y_{j}=r_{j}+\gamma Q^{\prime }\left(s_{j+1},\mu^{\prime }\left(s_{j+1}|\theta^{\mu^{\prime }}\right)|\theta^{Q^{\prime }}\right)$.13:     Update $\theta^{Q}$ by minimizing the mean quadratic error function based on (14).14:     Update $\theta^{\mu }$ by sampled policy gradient $\nabla_{\theta^{\mu }}J$ given by (16).15:     Update the target networks:16:     $\begin{array}{l} \theta^{Q^{\prime }}\leftarrow \delta \theta^{Q}+\left(1-\delta \right)\theta^{Q^{\prime }},\\ \theta^{\mu^{\prime }}\leftarrow \delta \theta^{\mu }+\left(1-\delta \right)\theta^{\mu^{\prime }}. \end{array}$17:   **end for**18:**end for**

### 3.3. Algorithm Analysis

The analysis of some issues, such as time delay and the computational complexity, are discussed for the proposed DIDDPG algorithm in this section.

#### 3.3.1. Time Delay Analysis

Due to the inherent features of wireless transmission, the time delay is an inevitable issue that needs to be addressed in the UAV formation control process. It is known from (6) that the follower’s state update is dependent on previous delayed control strategies due to the time delay. That is, the actor input in a sampling interval is given by

(19) $$c\left(t\right)=\left\{\begin{array}{cc} c_{j-1}, & j\Delta T<t\leq j\Delta T+\tau ,\\ c_{j}, & j\Delta T+\tau <t\leq \left(j+1\right)\Delta T. \end{array}\right.$$

**Figure 6:**
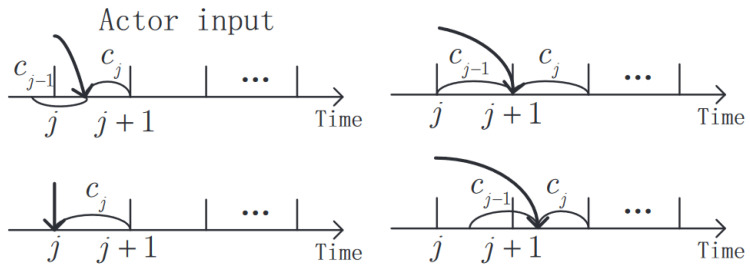
Different scenarios of actor input in a sampling interval.

The different scenarios of time delay on the actor input are shown as in Figure 6. It is necessary to further discuss the influence of delayed information on how to design the DIMDP. Below, two special cases are represented to show the effect of time delay on the actor input, state definition and state transition function design of DIMDP.

When τ=0, the actor immediately receives the control strategy, and there is no effect of the previous control strategy on the follower’s states. The discrete-time state update function is given by

(20) $$\begin{array}{c} z_{j+1}=Ez_{j}+D_{j}^{1}c_{j}+G_{j}. \end{array}$$

When τ=ΔT, the actor input only includes the previous control strategy in the *j*-th sampling interval, and the discrete state update function can be expressed as

(21) $$\begin{array}{c} z_{j+1}=Ez_{j}+D_{j}^{2}c_{j-1}+G_{j}. \end{array}$$

In fact, the time delay is influenced by many uncertainties, such as network topology, access technology and transmission channel quality, thus causing long and stochastic delays. Therefore, an arbitrary time delay should be further investigated, which is typically represented as τ∈[qΔT,(q+1)ΔT), and here *q* is a positive integer [48]. Then, based on (5) and (6), the relevant discrete-time state update function can be expressed as

(22) $$z_{j+1}=Ez_{j}+{\tilde{D}}_{j}^{1}c_{j-q}+{\tilde{D}}_{j}^{2}c_{j-q-1}+G_{j},$$

where

$$D_{j}^{1}=\int_{0}^{\left(q+1\right)T-\tau_{j}}e^{At}dtB,\;D_{j}^{2}=\int_{\left(q+1\right)T-\tau_{j}}^{T}e^{At}dtB.$$

When the arbitrary time delay is considered, the follower’s states are dependent on $c_{j-q}$ and $c_{j-q-1}$. Similar to (8), the state can be extended to be

(23) $$s_{j}=\left[{z_{j}}^{T},{c_{j-1}}^{T},{c_{j-2}}^{T},\cdots ,{c_{j-q-1}}^{T}\right].$$

Then, based on (22) and (23), the state transition function can be formulated as

(24) $$s_{j+1}=s_{j}{\tilde{F}}_{j}+a_{j}{\tilde{H}}_{j}+\left[G_{j}^{T},0_{3\times 1}\right],$$

where

$$\begin{array}{c} {\tilde{F}}_{j}=\left[\begin{array}{cccccc} E^{T} & 0 & 0 & 0 & \cdots & 0\\ 0 & 0 & 1 & 0 & \cdots & 0\\ 0 & 0 & 0 & 1 & \cdots & 0\\ \vdots & \vdots & \vdots & \vdots & \ddots & \vdots \\ {\left({\tilde{D}}_{j}^{1}\right)}^{T} & 0 & 0 & 0 & \cdots & 1\\ {\left({\tilde{D}}_{j}^{2}\right)}^{T} & 0 & 0 & 0 & \cdots & 0 \end{array}\right],\;{\tilde{H}}_{j}={\left[\begin{array}{c} 0\\ I_{3\times 3}\\ 0\\ \vdots \\ 0 \end{array}\right]}^{T}. \end{array}$$

The reward function can be defined as

(25) $$r_{j}=-\left(s_{j}\tilde{P}s_{j}^{T}+a_{j}Qa_{j}^{T}\right),$$

where

$$\tilde{P}=\left[\begin{array}{cc} \bar{P} & 0_{6\times \left(q+1\right)}\\ 0_{\left(q+1\right)\times 6} & 0_{\left(q+1\right)\times \left(q+1\right)} \end{array}\right].$$

Based on the above extension definitions of state, the state transition function and reward function for arbitrary time delays, the proposed DIDDPG algorithm can be similarly applied to address the UAV formation control problem with long and stochastic delays.

#### 3.3.2. Computational Complexity Analysis

In the following, the computational complexity, typically described as the floating point operations per second (FLOPS) of the training and validating processes for the proposed DIDDPG algorithm is investigated. In fact, the operation, such as multiplication and division, is regarded as a single FLOP. In the training process, the FLOPS can be derived as the computation times in actor and critic networks. In the validating process, only the main actor network needs to be considered because there is no replay buffer and critic network.

The computational complexity of the training process can be deduced as [49]

(26) $$\begin{array}{c} v^{activation}u_{i}+2\times \sum_{m=0}^{M-1}u_{m}^{actor}u_{m+1}^{actor}+2\times \sum_{n=0}^{N-1}u_{n}^{critic}u_{n+1}^{critic}\\ =O\left(\sum_{m=0}^{M-1}u_{m}^{actor}u_{m+1}^{actor}+\sum_{n=0}^{N-1}u_{n}^{critic}u_{n+1}^{critic}\right), \end{array}$$

where *M* and *N* are fully connected layers for the actor network and critic network, respectively. $u_{i}$ means the unit number in the *i*-th layer, and $v^{activation}$ determined by the activation layer’s type such that $v^{activation}=1$, $v^{activation}=4$ and $v^{activation}=6$ represent the Relu layer, sigmoid layer and tanh layer, respectively.

During the validation process, only the main actor network exists. Then, the computational complexity for the validation process is given by

(27) $$O\left(\sum_{n=0}^{N-1}u_{n}^{critic}u_{n+1}^{critic}\right).$$

In the proposed DIDDPG-based UAV formation algorithm, double fully connected layers with 30 units and 1 units, respectively, are used to build the actor network, and Relu and tanh layer are used as the activation layer. Double fully connected layers with 60 units and 1 units, respectively, are used to build the critic network, and the Relu layer is used as the activation layer. Based on (26) and (27), the computations of the actor network and critic network are obtained as 756 and 900, respectively.

## 4. Simulation Results and Discussions

Numerical experiments are presented in this section to evaluate the performance of DIDDPG algorithm. The flight data are designed based on real UAV flight data in [29,30]. First, we show the effectiveness and convergence of the proposed DIDDPG algorithm. Then, we compare proposed algorithm with existing algorithms for performance evaluation. Last, it is verified that the proposed optimal policies are applicable to long arbitrary time delays. As a case study, a typical 2D UAV formation with constant altitude is investigated.

In order to avoid collisions and improve the formation, the desired velocity and headway (i.e., the relative distance between the leader and the follower) are often influenced by each other. Typically, the expected headway needs to be adjusted in real time according to the UAV velocity change, that is, the expected headway will become larger with the increase in the desired UAV velocity. As an example for simulations, we set this relationship as a typical sigmoid function as [50]

(28) $$v\left(h\right)=\left\{\begin{array}{cc} 0, & 0<h<h_{\min}\\ \frac{v_{\max}}{2}\left(1-\cos\left(\pi \frac{h-h_{\min}}{h_{\max}-h_{\min}}\right)\right), & h_{\min}\leq h\leq h_{\max}\\ v_{\max}, & h>h_{\max} \end{array}\right.$$

where *h* denotes the headway, $h_{min}$ and $h_{max}$ represent the maximum and minimum headway, respectively, and $v_{max}$ means the maximum velocity.

In the simulations, the system parameter settings are presented as in Table 1.

### 4.1. Performance Comparison of Convergence

The convergence of the proposed DIDDPG algorithm is evaluated and analyzed under various reward function forms and learning rates, and time delay is uniform in [0,0.2ΔT]. In order to facilitate performance comparison, we take the following normalization measure to the cumulative rewards as

(29) $${\bar{G}}_{N}=\frac{G_{N}-G_{\min}}{G_{\max}-G_{\min}},$$

where $G_{\max}$ and $G_{\min}$ are the maximum and minimum cumulative rewards, respectively.

**Figure 7:**
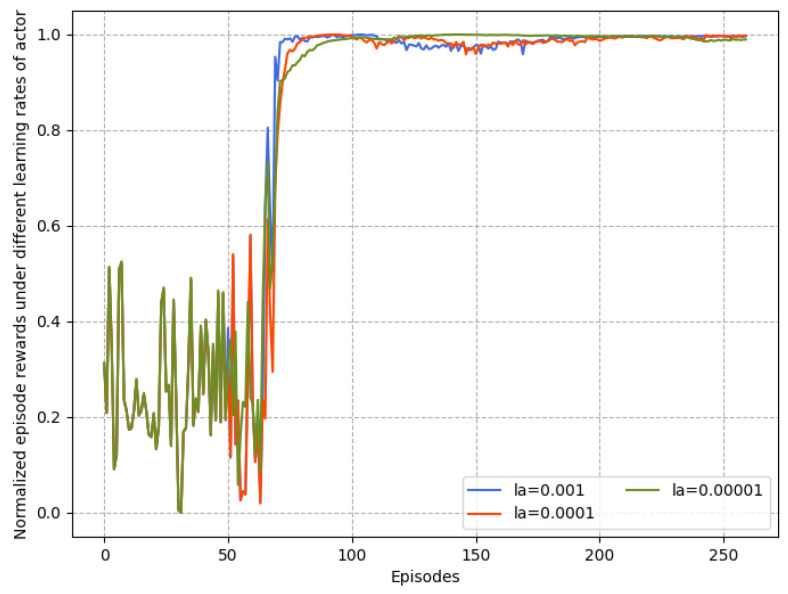
Normalized reward comparison with different learning rates of actor.

**Figure 8:**
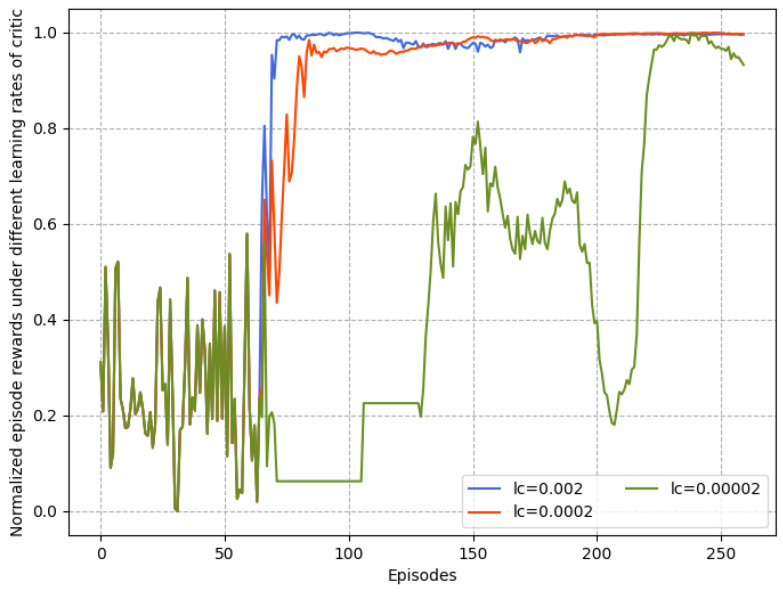
Normalized reward comparison with different learning rates of critic.

**Figure 9:**
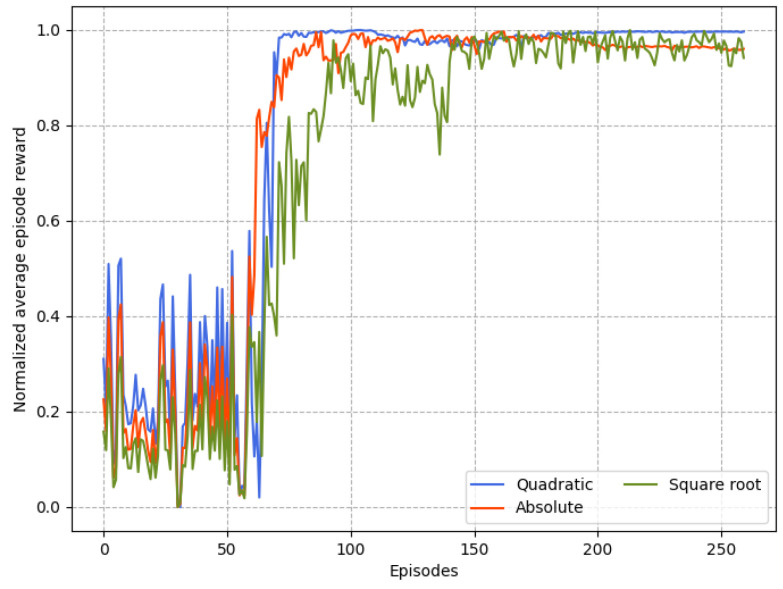
Normalized reward comparison with different forms of reward function.

Figure 7 and Figure 8 depicts the convergence of the proposed intelligent control algorithm under different actor and critic learning rates when the reward function is quadratic. If the learning rate is too small, the gradient descent could be slow, or the gradient descent may overshoot the minimum value such that it will fail to converge or even diverge. Obviously, in the case $l_{c}\;=\;0.00002$, the parameter update speed is slow, resulting in the inability to quickly find a good descending direction. Thus, the suitable range of values of $l_{a}$ and $l_{c}$ when the reward function is quadratic is obtained. In Figure 9, the effects of three types of reward functions under suitable learning rate values from Figure 7 and Figure 8 on the convergence performance of proposed algorithm are compared. It can be observed that the learning process of the case of quadratic reward function is the fastest and most stable. It indicates that, within suitable learning rates, the quadratic reward function consistently outperforms other forms and achieves the most benefit for the purposed intelligent control algorithm because it is consistent with the cost function of the UAV formation.

### 4.2. Performance Comparison of Different Scenarios

**Figure 10:**
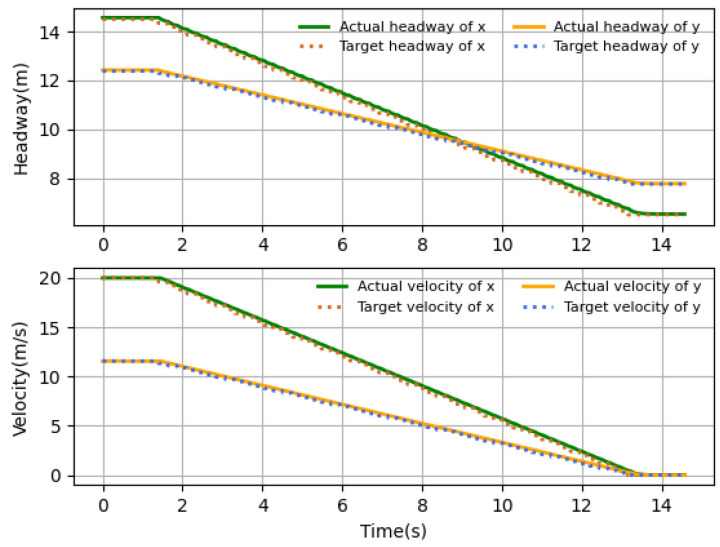
Harsh brake.

**Figure 11:**
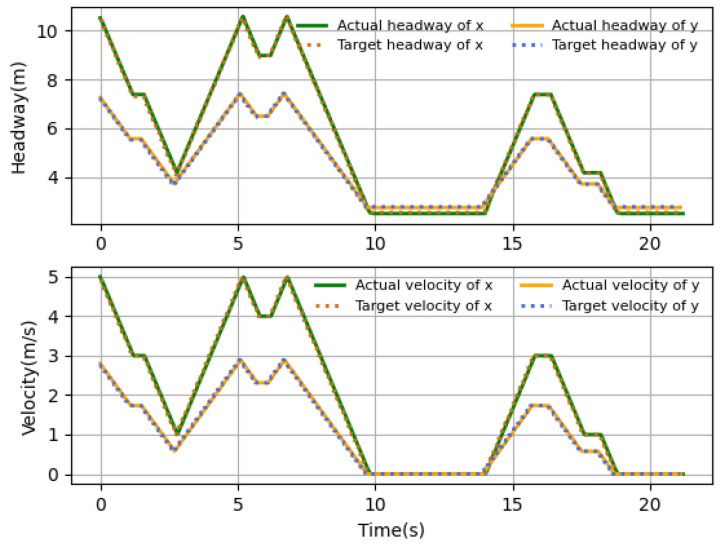
Stop-and-go.

**Figure 12:**
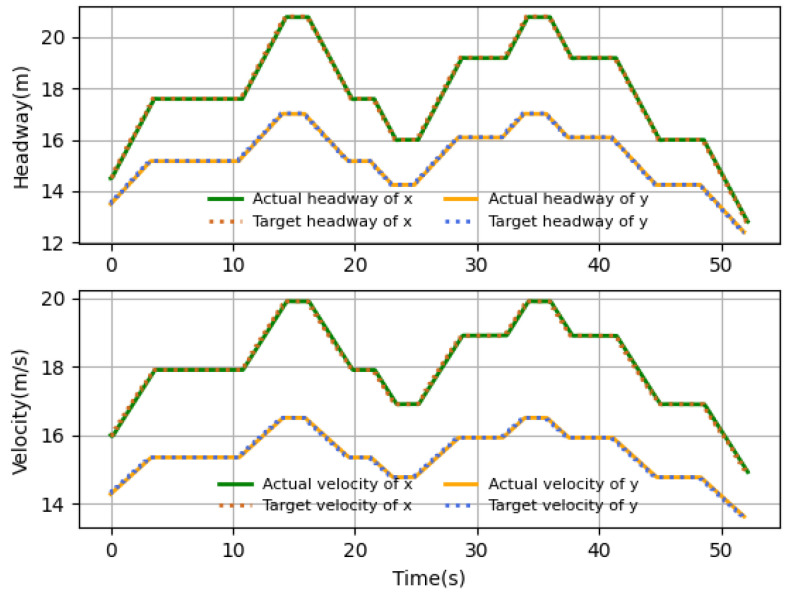
Speed limit.

The velocity and headway tracking performance in the presence of different time delays under different application scenarios are shown in Figure 10, Figure 11 and Figure 12. In the simulations, three scenarios are considered, including harsh brake, stop-and-go and speed limit. These three basic cases are covered by most application tasks, and the proposed algorithm has good practicality if it can satisfy the control requirements in these three cases. The simulation results show that the follower can track the desired states accurately by the proposed algorithm. Figure 10 shows the case when the follower suddenly meets an obstacle and needs to brake harshly, and the rapid velocity decline happens to represent the harsh brake. It takes about 11 s for the follower to stop from 20 m/s. Figure 11 shows the application scenario when UAV needs to stop and hover sometimes. For example, the UAV-assisted wireless powered IoT network, where UAVs hover to visit IoT devices and collect data, and the velocity variations are typically small. It can be seen that near 10 s, the velocity reaches the desired value and the headway stops changing; although there is a small error between the desired states, it is still within the acceptable range, and at 14 s when the follower starts flying, it can quickly follow the desired state. Figure 12 shows the case that UAV flights in restricted environments and the velocity change are limited. What is more, the headway’s tendency over time is the same as that of velocity, which is consistent with the relationship of the headway and velocity. The results show that proposed intelligent algorithm could be applicable to either high- or low-velocity cases and also could be used in large and small velocity variation conditions. In general, the proposed algorithm can derive the control strategy satisfying the tracking assignment under the above three common scenarios.

### 4.3. Performance Comparison with Different Aspects

**Figure 13:**
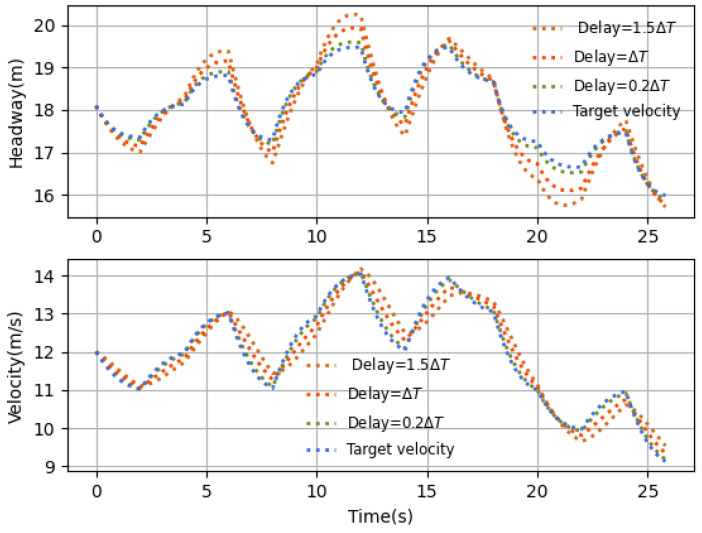
Velocity comparison with different delays.

**Figure 14:**
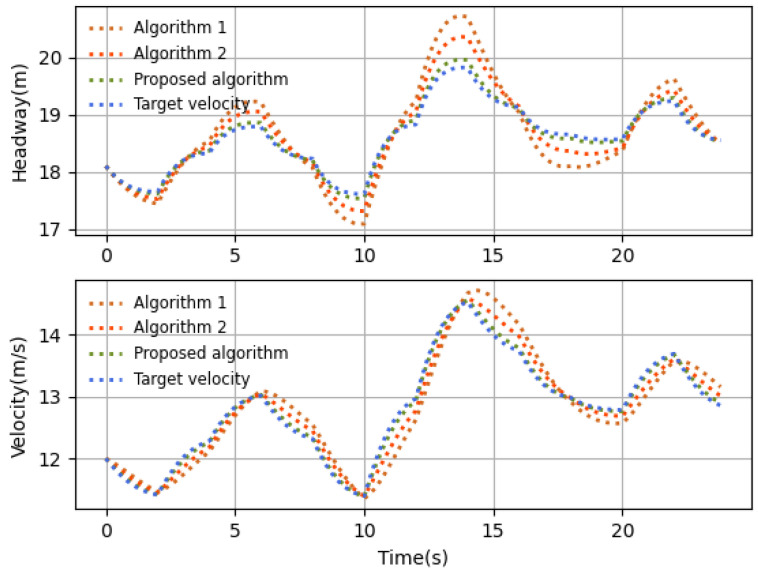
Velocity comparison with existing algorithms (Algorithm 1 in [39] and Algorithm 2 in [43]).

The performance comparisons with different time delays and existing algorithms are shown in Figure 13 and Figure 14, respectively.

In Figure 13, the time delay is set to be 0.2ΔT, ΔT and 1.5ΔT, and here the deterministic time delay settings represent the three delay scenarios discussed in Section 3.3.1 to demonstrate the influence of time delays on the relative performance of the proposed algorithm. Figure 13 shows that a larger time delay leads to more serious performance degradation. For example, when the time delay τ=1.5ΔT, the control strategy executed in each sampling interval is the delayed control strategy but not the current control strategy, thus causing the followers to react slower. Fortunately, the control performance still meets the tracking requirement. It indicates that the proposed algorithm can effectively regulate the follower to achieve the stable tracking under various time delays. When the time delay τ=0.2ΔT, the follower can keep close to the desired states all the time, which indicates that the proposed algorithm can compensate for the effect of the time delay and improve the control performance.

Figure 14 shows that our proposed algorithm has the quickest response and best control performance compared with the existing works under the time-varying leader velocity. In the simulations, the sampling interval is ΔT=0.2 s, the time delay is uniform in [0,0.2ΔT], and the other system parameter settings are the same as those in Table 1. Actually, the existing algorithm in [39] does not include the previous actions into the state, which may lead to the insufficient utilization of the delay information. Therefore, although this existing algorithm can reach the desired states, it still reacts more slowly. The existing algorithm in [43] does not consider the latency information in the agent environment, resulting in its performance being worse than the others.

## 5. Conclusions

UAV formation can be deployed in a multitude of surveillance scenarios. The leader–follower approach can effectively improve the efficiency of the whole formation. Since the desired velocity and time delay are dynamic due to different scenarios and the inherent feature of wireless communications, it is taken into account in the optimization formation problem in this paper. In order to compensate for the effect of time delay, a new MDP, called DIMDP, is designed by including previous actions into the state and reward function, and then the DIDDPG algorithm is proposed to solve the DIMDP of the UAV formation. The reward function form is designed, dependent on the quadratic cost function relevant to the objective of the optimization formation problem. After training, the intelligent control strategy can be derived for the follower. The simulation experiments demonstrate that the proposed intelligent controller can effectively alleviate the effects of time delays and is applicable to high dynamic formation scenarios. Compared with existing DRL algorithms with or without time delays, the proposed DIDDPG algorithm can achieve better control convergence and stability. However, the proposed algorithm is designed based on the flight data in the simulation according to the existing literature, and the lack of the real-world data or realistic simulation environments needs to be addressed in future work. The cooperative formation control system considered and designed in this paper aims to achieve control of the entire formation by dividing it into individual units and realizing the tracking control of each LF unit. However, at present, the construction of the multi-UAV cooperative control system and the research on multi-objective control algorithms are gradually attracting attention, and the multi-intelligent reinforcement learning algorithm can be studied in the future.

## Figures and Tables

**Table 1:** Simulation parameter settings.

Symbol	Description	Setting
$S^{v}$	Velocity space	[0,30] m/s
$S^{p}$	Acceleration space	[−5,5] m/s${}^{2}$
*K*	Mini-batch size	32
*N*	Episode	260
*M*	Time steps	200
$l_{a},\;l_{c}$	Learning rates for actor and critic	0.001, 0.002
γ	Discount factor	0.97
$h_{max}$	Maximum headway	30
$h_{min}$	Minimum headway	5
ΔT	Sampling interval	0.2 s

## Data Availability

The data presented in this study are available on request from the corresponding authors.
